# Systematic review of 3D-printed surgical devices for austere environments: an evaluation guideline

**DOI:** 10.3389/fmedt.2026.1908227

**Published:** 2026-09-02

**Authors:** Kyung-Hoon Moon, Matthew Cant, Mehdi Saeidi, Arul Ramasamy, Anthony M. J. Bull

**Affiliations:** 1 Department of Bioengineering, Imperial College London, London, United Kingdom; 2 Academic Department of Military Trauma & Orthopaedics, Research & Clinical Innovation, Defence Medical Services, Birmingham, United Kingdom

**Keywords:** 3D printing, additive manufacturing, austere environment, evaluation guideline, surgical devices

## Abstract

**Background:**

Three-dimensional (3D) printing is a common manufacturing technique. It is being adopted to produce essential surgical equipment in austere settings around the world. However, the evaluation of these products remains inconsistent. This raises safety concerns and limits translation to clinical use. To address this, this systematic review aims to identify 3D-printed surgical devices intended for austere environments and examine their evaluation methodologies. The findings will inform the development of an evaluation framework that supports the design of patient-safe devices.

**Methods:**

Medical and engineering bibliographic databases were searched to identify literature describing 3D-printed surgical devices for use in austere environments. Two independent blinded reviewers screened articles in two stages using abstracts and full texts. The review was conducted in accordance with PRISMA guidelines. In line with the EU Medical Device Regulation, device evaluations were categorised into manufacturing specifications, mechanical performance, sterilisation, biocompatibility, and usability. Reported methods of device evaluation were extracted and compared against the relevant published standards.

**Results:**

15 studies met the inclusion criteria. The austere environments included low-middle income countries, military settings, and space exploration. 13 out of 15 studies used Fused Deposition Modeling printers. All materials were thermoplastic. Printed equipment ranged from external fixators to surgical sets. The printing details were well reported. However, there was inconsistency in testing methods, resulting in data that is incomparable across the studies. 2 of 15 papers conducted mechanical testing for external fixators using American Society for Testing and Materials standards but used different parameters. 2 studies used self-developed mechanical testing protocols. 7 of 15 papers reported usability analysis using various methodologies. 3 of 15 papers described testing for sterilisation of the equipment, but only one paper evaluated its effect on the device. There was no biocompatibility testing.

**Conclusion:**

Despite the potential implications for patient safety, all 15 studies showed considerable variability in device evaluation methods. To address this, this review recommends adopting established standards from ISO, IEC and ASTM International. The proposed framework covers the key domains: manufacturing specifications, mechanical performance, sterilisation, biocompatibility and usability. Implementation of this structured approach may enhance standardisation and support the development of safer devices.

## 1 Introduction

Across austere environments, such as conflict zones and low- and middle- income countries (LMICs), surgical capacity is severely stretched by medical device shortages (1, 2). Many Western-developed devices remain unaffordable, while in-country production is constrained by a shortage of skilled labour. Consequently, there is a growing imperative to develop low-cost, reusable surgical devices that can be easily manufactured locally with minimal training (3, 4).

A potential solution is the utilisation of 3D-printing techniques to manufacture surgical devices. This type of additive manufacturing has been widely used for surgical innovation since the 1990s for better ergonomics and customised implants (5–11). Metal 3D-printed (3DP) surgical devices are produced in high-resource settings, requiring costly equipment and extensive post-processing. This is not well-adopted in austere environments (12). In contrast, thermoplastic 3DP has become more affordable, reliable and easier to operate (13). Over the last two decades, desktop 3D printer costs have fallen by more than 100-fold, while layer height has improved from 1 mm to 0.1 mm (14, 15). Ruggedised military printers are already in use to produce spare parts in deployed areas (16, 17). Thermoplastic 3DP is, therefore, a potential method of manufacturing surgical devices at the point-of-need in austere environments.

Researchers have explored using 3D printing for medical devices intended for austere environments. Wong et al. (18) and Kondor et al. (19) reported developing basic surgical instrument sets for use in space exploration and deployed military contexts, respectively. Wisdom et al. (20) successfully produced a laryngoscope using a ruggedized 3D printer in an Arctic environment. The field is now transitioning from proof-of-concept studies toward productisation. This shift necessitates robust and standardised evaluation frameworks to ensure patient safety, reproducibility, and eventual regulatory compliance. The absence of standardised data limits comparability between studies and risks the development of suboptimal devices that may compromise patient safety (21, 22). The establishment of standardised testing protocols for 3D-printed surgical devices is now essential.

**Figure 1:**
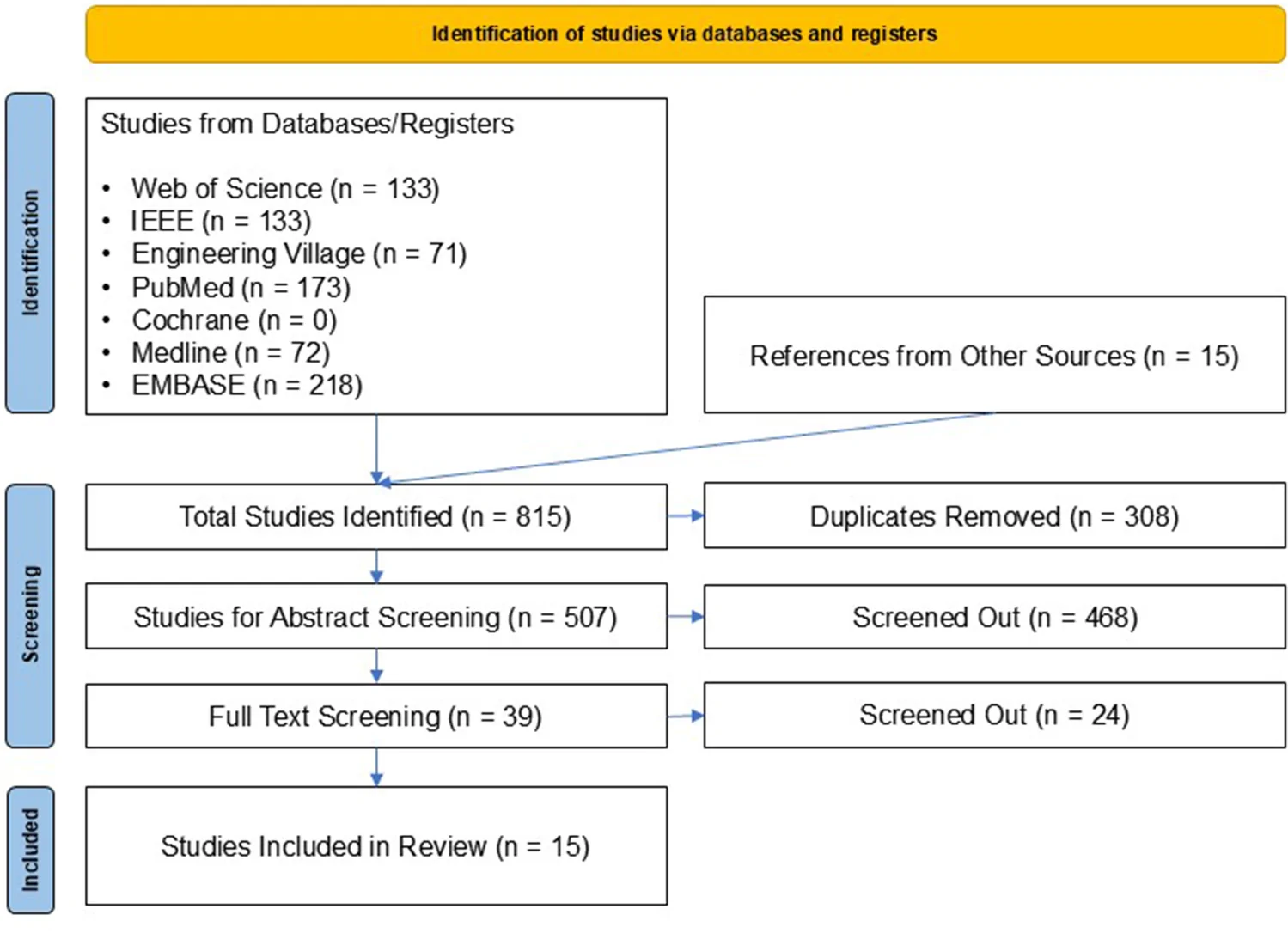
Flow diagram of the identification and inclusion of papers that reported on 3D-printed surgical devices for use in austere environments.

**Figure 2:**
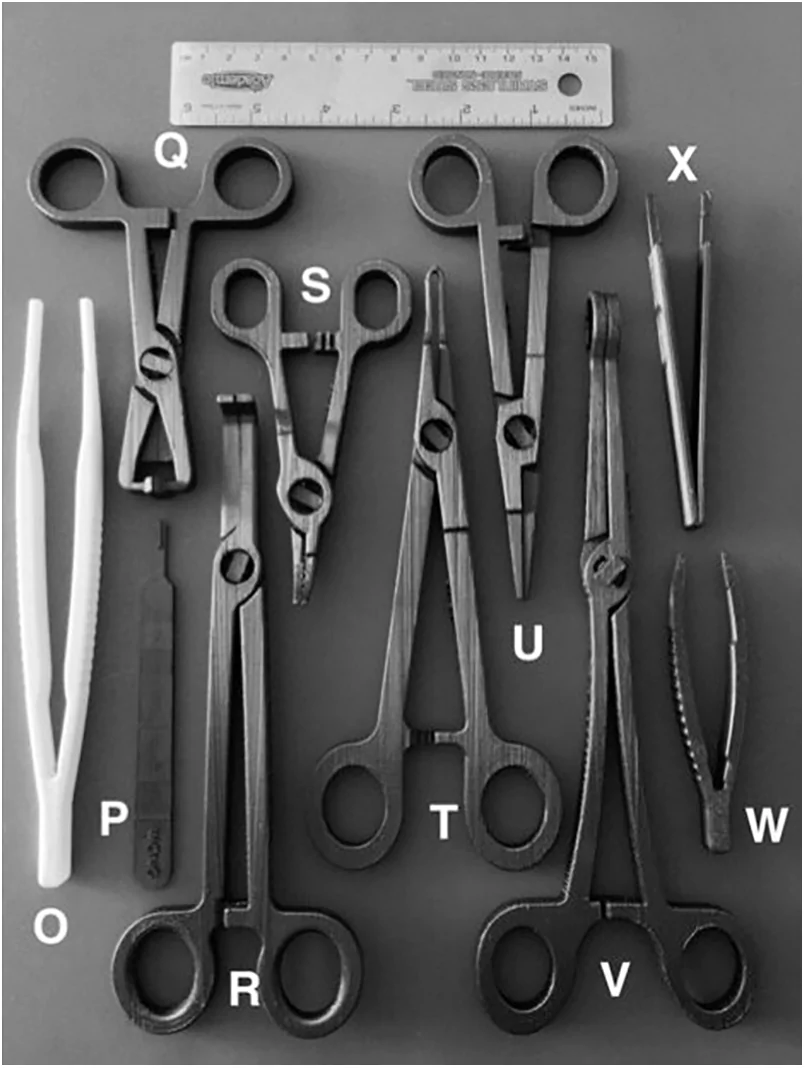
3D-printed surgical instruments by Wong et al. (39) (O) tissue forceps, (P) scalpel handle, (Q) towel clamp, (R) right angle clamp, (S) curved haemostat, (T) tissue clamp, (U) straight haemostat, (V) sponge stick, (W) smooth forceps, and (X) toothed forceps. Produced by Wong et al. with permission from the Journal of Aerospace Medicine & Human Performance, Aerospace Medical Association.

**Figure 3:**
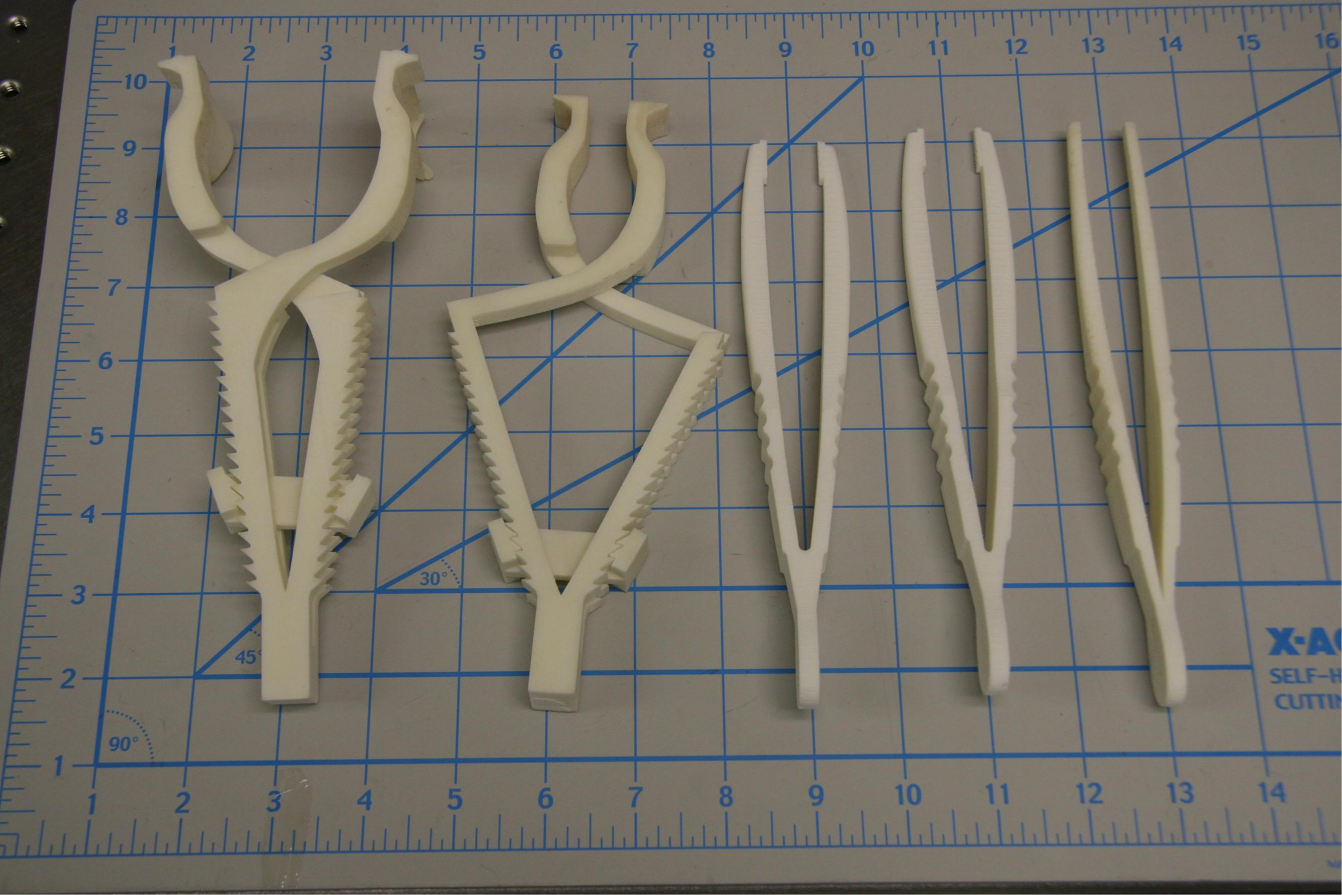
Picture showing two types of 3D-printed self-retaining retractors and three different designs of forceps by George et al. (35, 36) Source: Images copyright Dr. Mitchell George. Used with permission.

**Figure 4:**
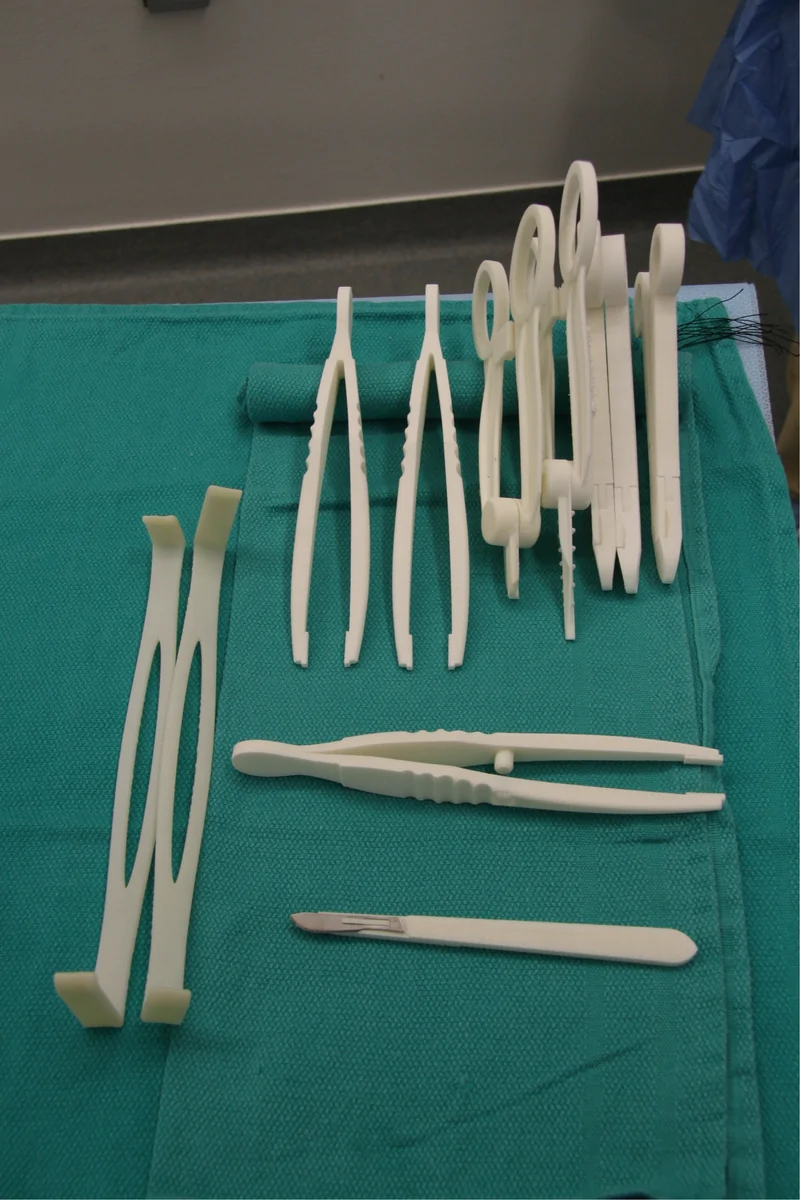
Picture showing 3D-printed army-navy retractors, forceps, scissors, needle holders and a scalpel handle by George et al. (35, 36) Source: Images copyright Dr. Mitchell George. Used with permission.

**Figure 5:**
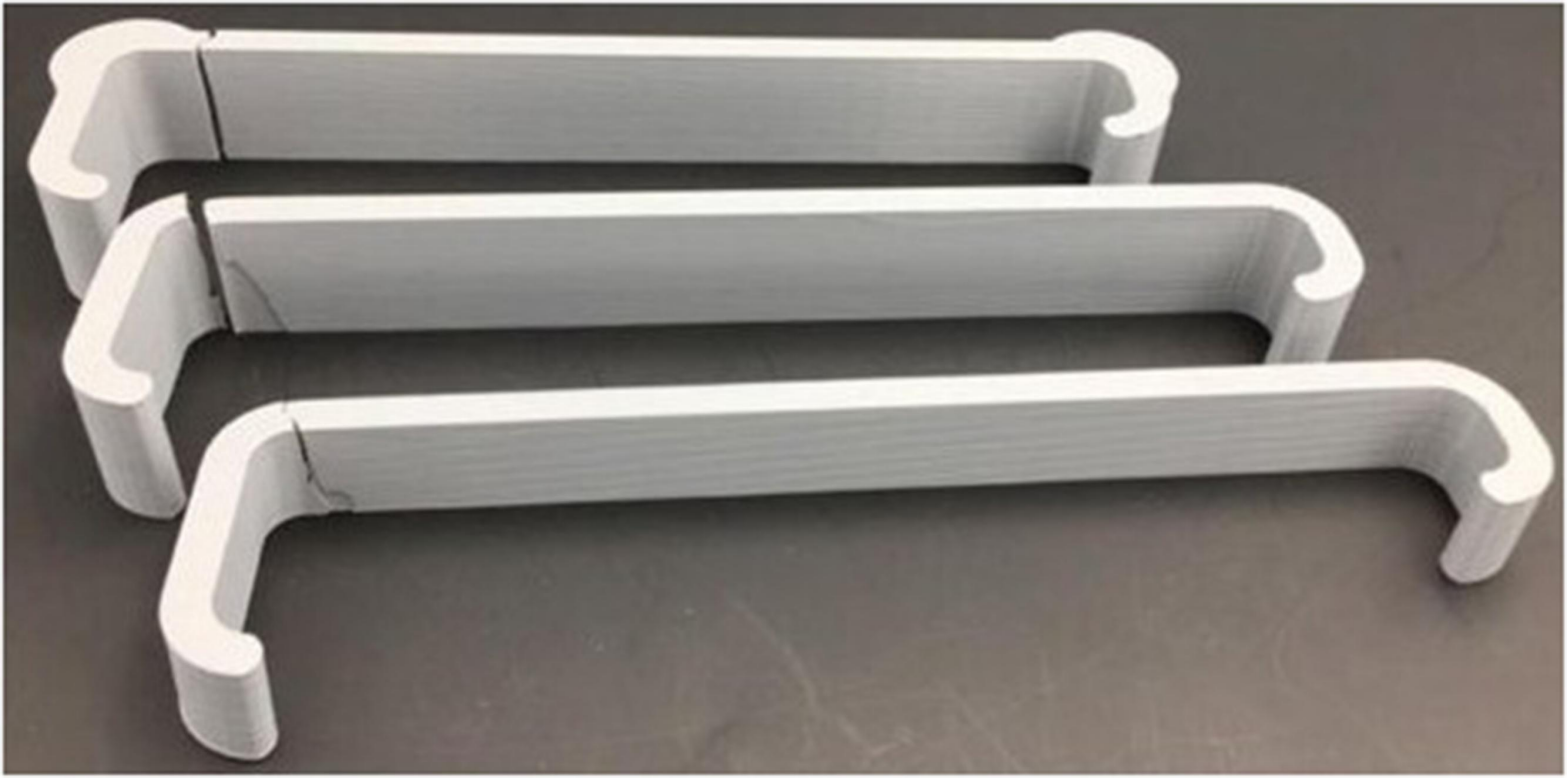
Broken retractors after mechanical testing by Chen et al. They were designed to have reinforced joints and increased width. Source: Chen et al. (12). License: Creative Commons Attribution 4.0 International License.

**Figure 6:**
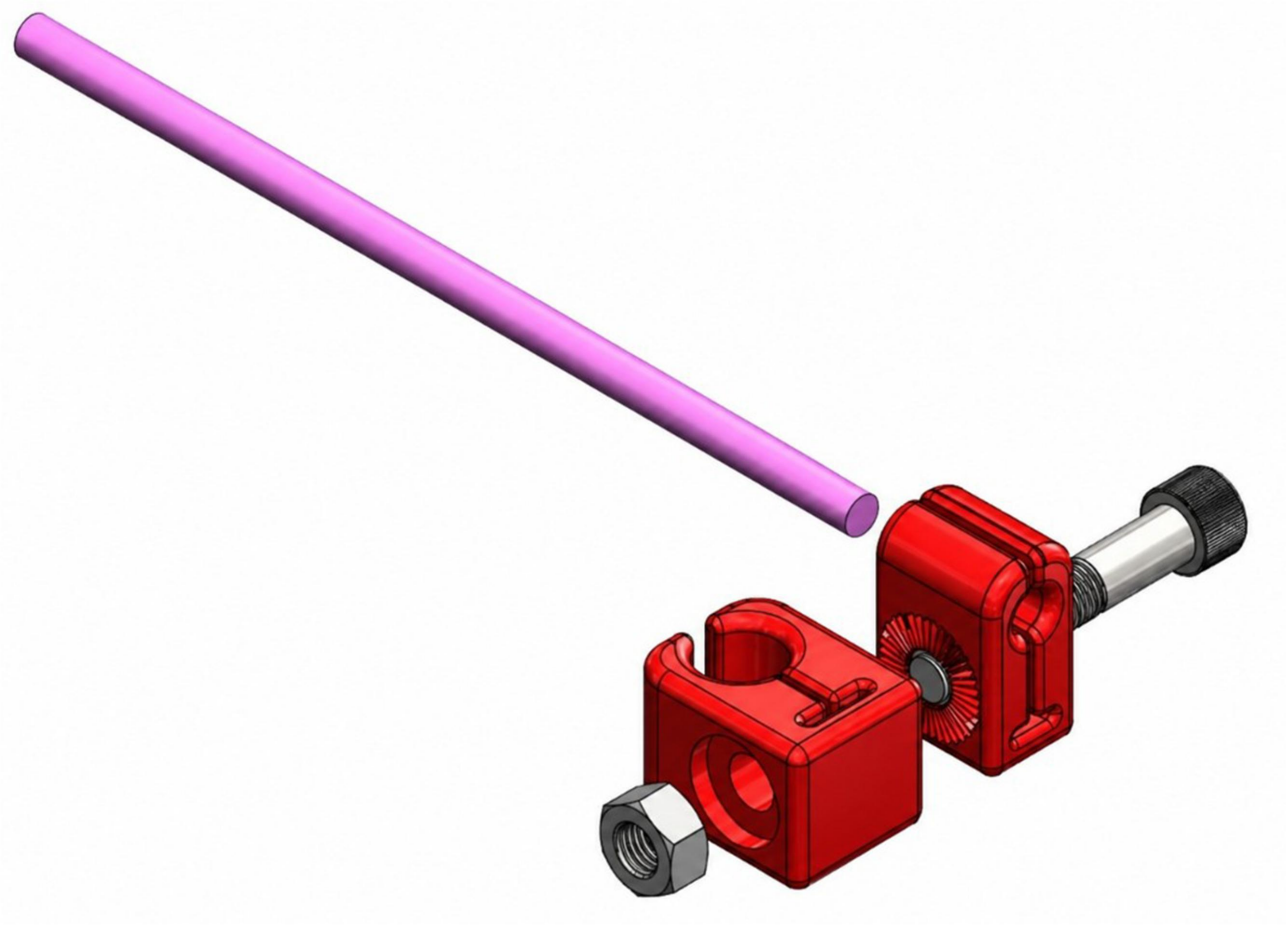
Exploded view of the 3D-printed external fixator by Landaeta et al. (32). License: Creative Commons Attribution 4.0 International License.

**Figure 7:**
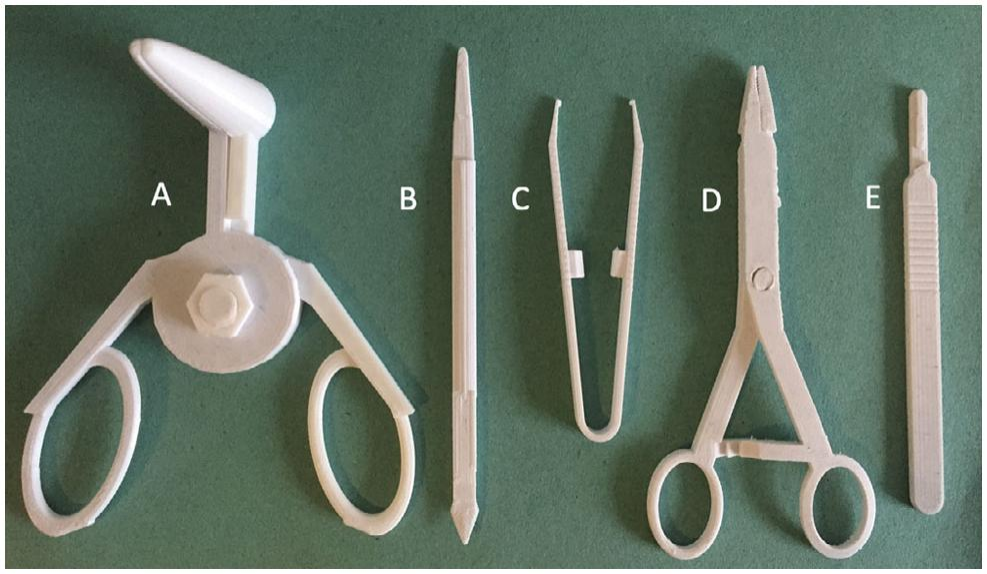
3D-printed ENT instruments by Zaidi et al. (41). **(A)** Killian's speculum, **(B)** Cottle/Freer elevator, **(C)** toothed forceps, **(D)** needle holders, and **(E)** scalpel handle. License: Creative Commons Attribution 4.0 International License.

**Figure 8:**
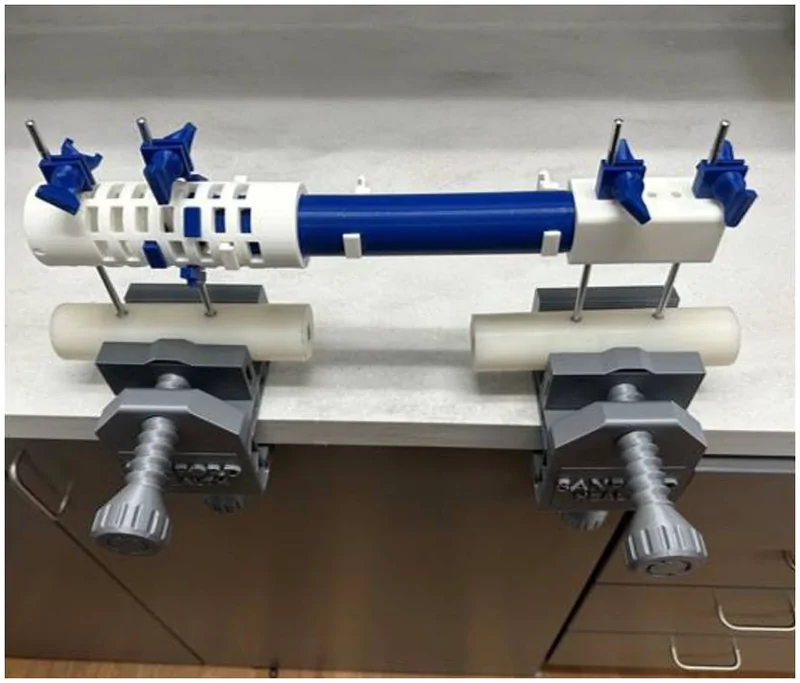
3D-printed external fixator by Skelley et al. (41). License: Creative Commons Attribution 4.0 International License.

**Figure 9:**
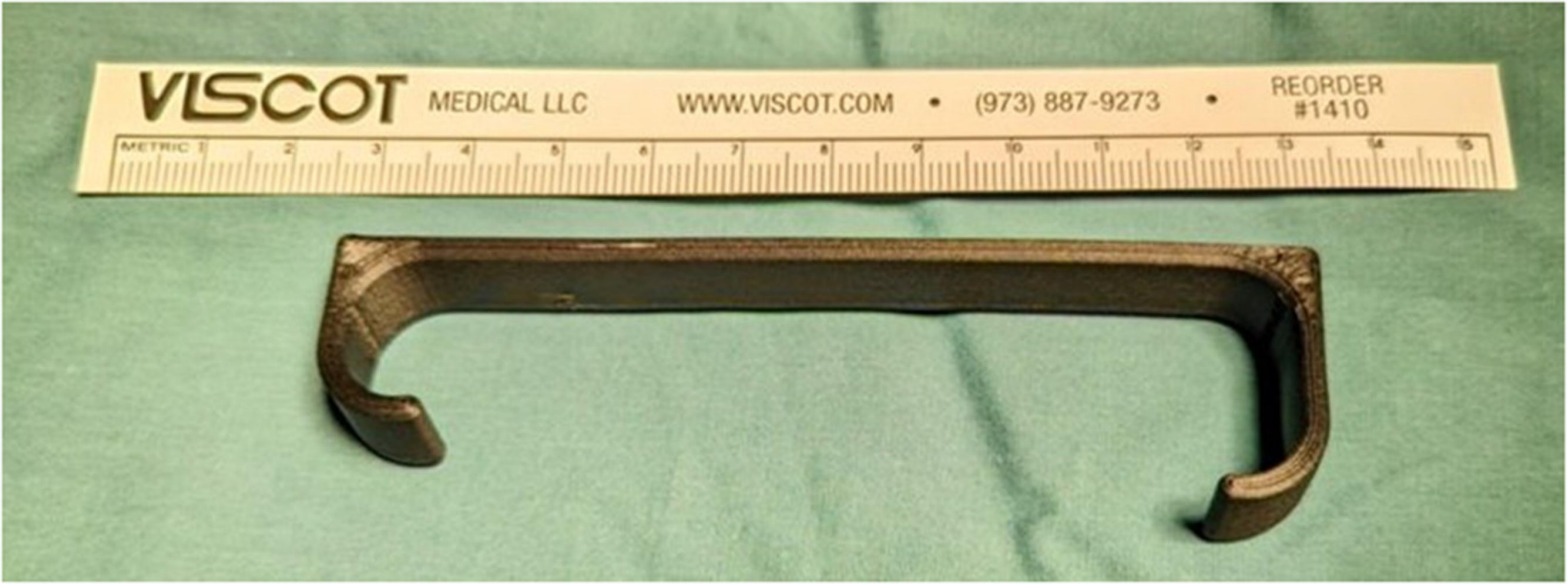
3D-printed Army-Navy retractor by Maxwell et al. (29). Source: Images copyright permission granted by co-author Mr David Becerra by private communication.

For conventional surgical devices, manufacturers operate within established regulatory frameworks and generate evidence in accordance with recognised standards. This is typically guided by established standards from the International Organisation for Standardisation (ISO) and the American Society for Testing and Materials (ASTM International). In cases where device-specific standards are unavailable, evaluation methodology is developed in accordance with overarching regulatory principles using risk analysis such as ISO 14971 (23). Regulatory frameworks such as Annex II Section 3 of the EU Medical Device Regulation (EU MDR) (24), the FDA Quality System Regulation 21 (25), Centre for Disease Control and Prevention (CDC) (26), and World Health Organisation (WHO) (27) outline the key domains of evidence generation. They are: manufacturing processes, mechanical performance, sterilisation, biocompatibility and usability. Together, these domains provide a structured framework for assessing device safety and performance. They offer a relevant basis for evaluating emerging 3D-printed surgical devices in austere environments.

This systematic review will identify and characterise 3D-printed surgical devices developed for use in austere environments. The method of evaluation will be critically appraised for its safety and performance across key domains, and will include commentary on its relationship to established regulatory frameworks. The findings will be used to make recommendations to strengthen patient safety and accelerate the responsible advancement of research in this field.

## 2 Methodology

This systematic review was conducted in accordance with the Preferred Reporting Items for Systematic Reviews and Meta-Analysis (PRISMA) 2020 guidelines (28). A protocol was developed to define the search strategy, eligibility criteria, and analytical framework. This was registered to PROSPERO under ID CRD420251250745. All methodological steps were predefined to minimise bias and enhance reproducibility.

### 2.1 Search strategy

A comprehensive literature search was conducted from database inception to 17 May 2026 across the following databases: EMBASE, MEDLINE, Pubmed, Cochrane Library, Web of Science, IEEE Xplore, ASME Digital Collection, and Engineering Village. To ensure coverage of grey literature and emerging technologies, Google Scholar and institutional thesis repositories (DART-Europe, ProQuest, British Library EThoS, and Imperial Spiral) were also searched. Search strategies were tailored to each database using a combination of MeSH terms and free-text keywords. Boolean operators (AND, OR) and truncation were applied to maximise sensitivity. The search strategy combined three core concepts: three-dimensional printing, austere environments, and surgical devices. A full reproducible search strategy for each database is provided in Table 1.

**Table 1:** Example of a search string key words with boolean operators. Each Strings were combined using Boolean operation “AND”.

Search strings	Key words
String 1	exp three dimensional printing/ or three-dimensional print*.mp. 3d print*.mp. 3d-print*.mp. 3-dimensional print*.mp. additive manufacture.mp. additive manufacturing.mp.
String 2	warfare.mp. or exp warfare/ exp war/ wars.mp. armed conflict.mp. military.mp. military medicine.mp. or exp military medicine/ army.mp. or exp army/ navy.mp. or exp navy/ naval medicine.mp. or exp naval medicine/ air force.mp. or exp air force/ aerospace medicine.mp. or exp aerospace medicine/ exp natural disaster/ or natural disaster*.mp. disaster medicine.mp. or exp disaster medicine/ austere environment.mp. austere setting*.mp. exp resource limited setting/ or resource-limited.mp. exp developing country/ or developing countr*.mp. (low or middle income countr*).mp. exp low income country/ or exp middle income country/ or LMIC.mp.
String 3	surgical equipment.mp. or exp surgical equipment/ surgical device*.mp. surgical instrument*.mp. orthopaedic equipment.mp. or exp orthopedic equipment/ orthopaedic instrument*.mp. orthopaedic fixation device*.mp. or exp orthopedic fixation device/ exp medical device/ or medical device*.mp. medical equipment.mp.

### 2.2 Eligibility criteria

Studies were included if they met the following criteria: (A) original research describing the design or production of a novel surgical device or instrument, (B) the device is intended for contact with sterile tissue or the vascular system, (C) the device is explicitly intended for, or reasonably applicable to, use in austere or resource-limited environments, and (D) the device is manufactured using thermoplastic 3D printing techniques. Austere environments were defined as settings characterised by constrained infrastructure, limited resources, disrupted supply chains, or extreme environmental conditions. These include, but are not limited to, low- and middle-income countries, conflict zones, disaster settings, and remote or space environments. Studies were excluded if they: (A) did not involve surgical devices or instruments, (B) did not describe a novel device or manufacturing process, (C) were review articles, editorials, or conference abstracts without full data, and (D) focused solely on non-clinical tools (e.g., anatomical models without intraoperative use).

### 2.3 Study selection

All identified records were imported into Rayyan (Rayyan Systems Inc., Cambridge, MA, USA) for deduplication and screening. Two independent reviewers (KHM and MC) screened studies in two stages: (1) title and abstract screening, and (2) full-text review. Disagreements were resolved through discussion and consensus. Where necessary, a third reviewer (AR) was available for arbitration. Inter-reviewer agreement was assessed during the pilot screening phase to ensure consistency in applying eligibility criteria.

### 2.4 Data extraction

A standardised data extraction form was developed. Extracted data included study characteristics (e.g., year, country and setting), device type and intended application, 3D printing technology and materials, manufacturing parameters and post-processing, evaluation methods across predefined domains, key findings and reported limitations.

### 2.5 Quality assessment and risk of bias

Given the heterogeneity of study designs and the predominance of early-stage engineering and proof-of-concept studies, a modified qualitative appraisal approach was adopted. Five key domains were derived from FDA and EU MDR regulations in the development of novel surgical devices. These are manufacturing, mechanical testing, sterilisation, biocompatibility, and usability. Studies were assessed across five domains relevant to medical device evaluation. Each domain was evaluated for methodological rigour, completeness of reporting, and alignment with recognised standards (e.g., ISO or ASTM). Studies were not excluded based on quality; however, methodological limitations were explicitly considered in the synthesis and interpretation of findings. This approach was chosen in place of conventional risk-of-bias tools (e.g., ROBINS-I), which are not well suited to early-stage device development studies lacking clinical outcomes.

### 2.6 Data synthesis

Due to heterogeneity in study design, device type, and outcome measures, a quantitative meta-analysis was not feasible. A structured narrative synthesis was conducted. Findings were organised according to the five predefined evaluation domains, enabling systematic comparison across studies.

(1) Manufacturing was assessed in terms of reproducibility, with attention to the reporting of printing time and material specifications. (2) Mechanical testing was appraised based on the use of quantifiable, standardised, and comparable performance metrics. (3) Sterilisation domain was evaluated with respect to the method employed, its justification, and its impact on device integrity and function. (4) Biocompatibility assessment examined whether studies addressed the safety of device–tissue interaction in a surgical context. (5) Usability was assessed for clinical relevance, methodological rigour, and alignment with validated evaluation frameworks.

Within each domain, studies were analysed in relation to three areas. (1) Use of standardised or validated methods, (2) Reproducibility of evaluation, and (3) Alignment with standards and regulatory frameworks, including ISO and ASTM standards, EU MDR and FDA Quality System Regulation. This framework-based synthesis was selected to benchmark current practices against established medical device evaluation principles and to identify critical gaps in translational readiness.

## 3 Results

In total, 800 records were identified by the database searches, and 15 grey source references were added. 308 duplicates were detected and removed. 15 of these met the inclusion criteria (Figure 1).

Eleven of the fifteen (11/15) studies were conducted by nine US teams (19, 29–38). Three (3/15) studies were conducted by one Canadian team (18, 39, 40). One (1/15) study was from the UK (41). Table 2 summarises these findings with relevant figures. The Canadian publications were related to the progressive development of 3D-printed surgical instruments. Their aim was the potential use in the future manned space missions to Mars (an austere space environment). Four papers were described as point-of-need additive manufacturing in the military domain (19, 29–31). Five papers referred to the potential use of their products in LMIC austere environments. Two were aimed at all three domains of military, LMIC and space (35, 36). Four of these author groups (18, 19, 37, 39–41) produced “Sets” of instruments.

**Table 2:** 3DP surgical devices for austere settings denoting details of mechanical testing, usability testing and sterilisation.

Reference	Application Purpose	Instruments	Printing Location	Printer Type	Printing Time (hh:mm)	Material	Mechanical Tests	Usability Evaluation	Sterilisation
Kondor et al. (19)	Military	Army-Navy Retractor, Tissue forceps, Scalpel handle, Needle Driver (2 parts), Metzenbaum scissors (2 parts), Kelly haemostat (not in picture)	Lab Field Hospital (Only Kelly haemostat was printed in the field hospital)	FDM	1–3 h each	ABSi-Ag (ABS resin with silver loading)	—	Tasks on Chicken. Tasks on a wearable surgical simulation suit	Chemical
Rankin et al. (37)	LMIC	Army-Navy Retractor	Lab	FDM	0:40–1:40	PLA	Tests to failure	—	Autoclave
Wong and Pfahnl (18)	Space	Figure 2	Lab	FDM	Scalpel handle 0:25–0:51 Straight Haemostat 8:28 Curved Haemostat 7:26 Sponge Stick 10:24 Towel Clamp 9:05 Right angle clamp 9:50 Toothed forceps 1:58 Debakey Forceps 3:39 Smooth Forceps 1:40	ABS+ (ABS plus-P430 plastic)	—	Timed tasks in comparison to metal version by 13 surgeons. 5-point Likert scoring by 13 surgeons using a usability questionnaire.	—
Wong and Pfahnl (39)	Space	Figure 2	Utah Desert	FDM	As above	ABS+	—	Feedback from a surgeon	—
Wong (40)	Space	Figure 2	Lab	FDM	As above	ABS+	—	Timed task by 5 non-surgical analogue mars crew. Compared against using metal version	—
George et al. (35)	Military LMIC Space	Figure 3 Figure 4	Lab	SLS	Army-Navy 02:30 Scalpel handle 3:00 Straight Haemostat 5:00 Needle holder 6:00 Forceps 3:30	PP/ABS (Polypropylene and ABS)	—	3 ergonomic evaluations and 2 cadaveric hernia repair trials for development	—
George et al. (36)	Military LMIC Space	Figure 3 Figure 4	Lab		As above	As above	—	1 cadaveric hernia repair	—
Chen et al. (38)	LMIC	Figure 5	Lab	FDM	0:40–1:40	PLA	Tests to failure	—	—
Landaeta et al. (32)	LMIC	Figure 6	Lab	FDM	—	Nylon + CF (Nylon with chopped carbon fiber)	ASTM F1541	—	Autoclave
Chambers et al. (31)	Military	Retractor (invented)	Lab	SLA	—	Resin	—	—	—
Zaidi et al. (41)	LMIC	Figure 7	Lab	FDM	Scalpel Handle 0:13 Needle holder 0:47 Killian's speculum 02:13 Tooth Forceps 0:18	PLA	—	1 cadaveric septoplasty	—
Barnhill et al. (30)	Military	Scalpel handle, Haemostat	Sub-Sahara Desert	FDM	Scalpel 0:30	PAPC (Polyamide polyolefin with cellulose fibers)	—	—	—
Skelley (34)	LMIC	Figure 8	Lab	FDM	59:12	PLA	—	—	—
MacFadden et al. (33)	LMIC	Figure 8	Lab	FDM	As Above	PLA	ASTM F1541	—	—
Maxwell et al. (29)	Military	Figure 9	Warship	FDM	—	Nylon + CF	—	—	Autoclave

The 15 papers reported on 16 different types of surgical instruments and devices. They were external fixators, Army-Navy retractor, Cottle/Freer elevator, Kelly (curved) haemostat, needle driver, towel clamp, sponge stick, straight haemostat, curved haemostat, right angle clamp, Killian's speculum, Scalpel handle, Metzenbaum scissors, Adson's toothed forceps, Debakey tissue forceps, and Smooth forceps (Table 2).

### 3.1 Manufacture details

Three papers (3/15) printed their surgical instruments in austere settings—a military field hospital in Sub-Saharan Africa (30), onboard a naval ship (29), and desert research station (40). Ten (10/13) teams used Fused Deposition Modeling (FDM) printers, with the exception of Chambers et al. (31), Maxwell et al. (29) and George et al. (35) who used stereolithography apparatus (SLA) and selective laser sintering (SLS) printers. Materials used to print the surgical equipment varied and were: nylon with carbon-fibre mixture, polylactic acid (PLA), Polyamide Polyolefin and Cellulose (PAPC), acrylonitrile butadiene styrene (ABS) and Photopolymer resin for SLA printer. PLA and ABS were most frequently described by four and five papers, respectively.

### 3.2 Mechanical testing

Mechanical testing using published standards and comparing to “gold standard” devices is a common approach used in the literature. Four papers (4/15) (32, 33, 37, 38) presented mechanical testing methods and results. Rankin et al. (37) and Chen et al. (38) used different testing jigs to analyse the tensile strength of 3DP Army-Navy retractors. Rankin established the maximum tensile strength by applying weight to the retractor via a webbing until failure. Chen et al. used a manual force test stand with a force gauge to obtain test results, resulting in recommendations on both retractor design and 3D printing parameters.

Landaeta et al. (32) and MacFadden et al. (33) based their tests on the American Society for Testing and Materials Standard Specification and Test Methods for External Skeletal Fixation Devices (ASTM F1541) (42). Both performed the mechanical test of the 3D-printed external fixators using non-cyclic axial compression. MacFadden et al. (33) used a mainstream metal external fixator as a comparison. However, different testing parameters were used. For example, Landaeta et al. and MacFadden et al. set the distance from the bone analogue surface to fixator clamps at 25 mm and 42 mm, respectively. ASTM recommends this parameter to be set at 50 mm for long bones. These parameters are well-known factors in the stability of the external fixation construct (43).

### 3.3 Sterilisation

Three papers (3/15) discussed the sterilisation of surgical devices. Two assessed the physical effect of autoclave sterilisation. The 3D-printed army-navy retractor by Rankin et al. (37) was sterilised with a chemical solution. It was submersed in a chemical sterilant of 2.4% glutaraldehyde solution with a pH of 7.5 for 20 min at 25 °C in accordance with CDC guidelines for critical medical devices that are sensitive to heat-based steam sterilisation (26). Samples from this device were then tested for bacterial load by polymerase chain reaction amplification of the 16S rRNA gene to measure intact bacterial DNA. This specimen collected contained bacterial gene products.

Maxwell et al. (29) steam sterilised their Nylon 3D-printed surgical retractor onboard USS Wasp using an autoclave Getringe (Gothenburg, Sweden). The Army-Navy style retractor was sterilised at 275F and 45 PSI for 5 min and reported to be ’stable and deemed appropriate’. However, no further evaluation was presented. Landaeta et al. (32) used a steam sterilisation cycle with an autoclave (Sterilmatic STM-ED steriliser) for their 3D-printed external fixator clamps. It underwent an autoclave at 135 °C for 25 min followed by 15 min of cooling and drying back to room temperature. The team measured dimensions before and after this process using a calliper. They reported a decrease in the dimension of bar hole, clamp width, height and length by 2.6%, 0.2%, 1.7% and 0.3%, respectively. The authors reported that sterilisation did not affect overall clamp use. However, there was no mechanical test post-sterilisation.

### 3.4 Biocompatibility

None of the included papers discussed or referred to the biocompatibility of the printed surgical devices or materials used.

### 3.5 Usability

Seven studies (7/15) carried out usability assessments with various surrogates: animal tissues (19, 39, 40); human cadaver tissues (35, 36, 41); and non-biological materials (18, 19). Outcomes were captured in various ways, including time to complete a task (18, 39, 40); verbal and written feedback (18, 19, 35, 36, 39, 41); and Likert rating scales (39). Usability testing served primarily to provide evidence of efficacy and safety (18, 19, 35, 36, 39, 41), whereas others used this to modify, adjust and refine the devices (35), and describe their use cases (36, 41). There was no application of the principle of usability testing using established usability guidelines such as IEC 62366-2.

Surgical simulations, including cadaver trials, identified issues arising from the material choices, particularly relating to their thermoplastic nature. George et al. found that instruments absorbed fluid they came into contact with during the cadaveric surgical procedure (35). They also reported a moderate amount of slippage of the needle through the jaws of the needle holder and crossing over of the forceps tips while grasping an object (36). Kondor et al. reported that the scissors utilising scalpel blades as cutting blades did not engage, failing to cut tissue (21). Zaidi et al. reported that the tip of the Freer, which was 2.75 mm thick, would curve upward in high humidity (41).

## 4 Discussion

This systematic review demonstrates that 3D-printed surgical devices intended for use in austere environments are predominantly early-stage, proof-of-concept technologies with highly inconsistent evaluation practices. The central finding is the absence of a standardised, regulatory-aligned evaluation across all assessed domains. This highlights a stark contrast to the improving regulations and validation methods of 3D-printed medical devices in the developed countries (44, 45). There is already guidance for clinical trials of 3D-printed medicine for high resource healthcare systems (46). Collectively, these gaps limit reproducibility, hinder cross-study comparison, and constrain translation toward clinically deployable devices. In this discussion, we propose a structured evaluation and reporting framework for each domain. The key and critical aspect is the multidisciplinary team approach, involving engineers, technicians, scrub team, clinicians and others involved in the care of the patient.

### 4.1 Manufacture detail

Manufacturing details, particularly printer type and material selection, were generally well reported. However, key parameters affecting reproducibility were inconsistently described. Three studies (19, 37, 41) did not report printing time. Print settings, such as layer height and infill percentage, were inconsistently documented. These settings have a direct impact on mechanical performance and production efficiency (38). Post-processing steps, which can increase overall production time and complexity, were also reported inconsistently. For example, Maxwell et al. highlighted that SLS requires specialised powder cleaning infrastructure, limiting its feasibility in austere environments (29). To address these gaps, we propose a standardised reporting framework aligned with ISO 13485:2016 (47) and reflecting the EU MDR (24) and FDA 21 Code of Federal Regulations Part 820 (25). This guidance should take into consideration the time, material, and resource constraints inherent to austere settings (48). A detailed example is presented in Table 3.

**Table 3:** Proposed Pre-clinical evaluation and reporting guideline for 3D-printed surgical devices for austere environments—device manufacture details.

Printing details	Explanation
Printing time	This should be recorded in hours and minutes for comparison.
Printer	Both the brand and model should be recorded for comparison and reproducibility. Required electricity supply should also be recorded to compare energy consumption.
Printing setting (In-fill%, height, perimeter, etc.)	Printing mode should be recorded (e.g., Speed, Structural or Custom). Further details such as Infill Percentage, Layer Height, Supports and Perimeter Layers should be recorded as they have direct impact on the printing time and performance.
Material Filament Used (type & quantity)	The material, mixed composite and brand details must be recorded for accurate reproducibility. If pre-printing treatment, such as drying for Nylon-12 should be recorded with time required to complete the process.
Post-production process	The details of the support removal and the post-production processes should be described. The average post-production processing time recorded will enable comparison between the devices.

### 4.2 Mechanical testing

Insufficient mechanical testing led to performance issues which became apparent during late-stage usability assessments. Application of established standards, such as ISO 7153-1 (49) could have identified these issues prior to pre-clinical evaluation. For example, section 4.11 of ISO 7153-1 describes the hold-strength test for needle holders which may have detected the slippage reported by George et al. (36). Similarly, by performing the test stated in sections 4.3.3 and 4.3.4 of ISO 1753-1, the blade misalignment, observed by Kondor et al. (19), could had been identified. Building on these standards, we propose a framework linking appropriate mechanical tests to device categories to support safer and more consistent development (Table 4).

**Table 4:** Proposed Pre-clinical evaluation and reporting guideline for 3D-printed surgical device for austere environments—mechanical testing.

Type of device	Explanation and examples of relevant mechanical testing standards
External Fixators	ASTM-F1541
Linear Retractors	Currently, no testing standard exists. Consider using the methodology used by Chen et al. (38) and direct comparison to the metal counterpart. This example utilizes a strain gauge with hanging weight applied to the retractor to replicate the forces applied to the device during clinical use.
Scalpel Handle	Currently, no testing standard exists. Consider following the ISO 14971 guideline and direct comparison to the metal counterpart. Consider the functional requirements such as repeated application and removal of metal blades. Tests using cyclical forces onto the blades to simulate cutting and scraping motion should be considered.
Needle Holders	ISO 7554-3 (49)—section 4.11
Haemostats	ISO 7554-3 (49)—section 4.10
Self-retaining Retractors	Currently, no testing standard exists. Consider following the ISO 14971 guideline and direct comparison to the metal counterpart. Consider the forces of tissue tension acting on the retractor during a surgical procedure.
Scissors	ISO 7554-3 (49)—section 4.3.3 and 4.3.4
Forceps	Currently, no testing standard exists. Consider following the ISO 14971 guideline and direct comparison to the metal counterpart. Consider the force required at the tip to hold various tissues (skin, subcutaneous layers, etc) or target instrument (suture needle) as testing methodology.

Inconsistent adherence to established protocols also limits comparability between studies. This was most evident in the mechanical evaluation of external fixators. Landaeta et al. (32) and MacFadden et al. (33) referenced ASTM F1541 (42) and other methodologies (50, 51) but used different key parameters such as pin-to-pin and rod-to-bone distances. These introduced variabilities that directly affect construct stability (43). This undermined performance comparisons and the interpretation of the reported performance. Stricter adherence to established testing protocols is therefore essential.

For certain devices, no specific mechanical testing standards currently exist. George et al. (35) reported instability of an attached blade on a 3D-printed scalpel handle and a problem with the crossing over of the forceps tips. This highlighted a need for rigidity testing (36). However, ISO 7153-1 (49) does not define methods to assess these characteristics. In such cases, a risk-based framework, as outlined in ISO 14971 (23), should be applied to develop a relevant test. Researchers must give up-to-date and evidence-based reasons for their methodology. This approach is illustrated by Chen et al. (12), who used a manual force test stand with a force gauge to iteratively assess their 3D-printed Army–Navy retractor designs. These bespoke testing methods could be used alongside conventional devices as comparative benchmarks. Further work is needed to develop standardised and reproducible protocols for such evaluations.

### 4.3 Sterilisation

Sterilisation assessments were similarly underdeveloped. In austere environments, devices are reused, requiring repeated sterilisation cycles. Common methods used are steam sterilisation via autoclaves and liquid chemical sterilants (50, 51). In this review, although some studies reported devices undergoing autoclave or chemical sterilisation, the evaluations were largely limited to qualitative observations and dimension checks. None provided a comprehensive functional reassessment following sterilisation cycles. This represents a critical gap given that repeated sterilisation is a requirement in austere and resource-limited environments. Based on the CDC and FDA recommendations (25–27), we make recommendations on sterilisation compatibility for future studies of 3DP surgical devices (Table 5). This provides a standardised reporting of the feasibility of reusing the devices after sterilisation.

**Table 5:** Proposed Pre-clinical evaluation and reporting guideline for 3D-printed surgical device for austere environments—sterilisation.

Sterilisation details	Explanation
Type of sterilisation	Depending on the 3DP device, an appropriate sterilisation should be advised. This may be steam, liquid chemical or others that are accessible in the target austere environment.
Detail of sterilisation	The details of optimum sterilisation instructions applied should be described. For steam sterilisation, this will include: autoclave brand and name used, duration, temperature, pressure. If liquid chemical sterilant is used, the name, volume and concentration of the agent used should be reported with exposure duration and the temperature where the processing took place.
Post sterilisation assessment	This allows functional validation of compatibility post-sterilisation. This may be a repetition of the previous mechanical tests or usability tests. The number of sterilisations the device has undergone should be recorded.

Autoclaving, typically performed at 121–134 °C for 3–15 min (52), can cause permanent deformation in commonly used thermoplastics such as ABS, PLA, and PETG. Some studies suggest that 3D-printed thermoplastics may be sterile immediately after extrusion due to high processing temperatures (19, 37). This is not feasible for low-resource environments. Nylon-12 is more resistant to autoclave conditions (53), but its use in filament-based 3D printing requires a lengthy pre-printing dehydrating process. For heat-sensitive devices, the CDC recommends low-temperature sterilisation methods, including Liquid chemical sterilants, such as glutaraldehyde-based formulations. These agents can achieve sterility when adhering to strict parameters of concentration, contact time, temperature, and pH. In extreme conditions, improvised approaches have also been reported. Simon et al. described the use of formaldehyde generated from paraformaldehyde tablets for instrument sterilisation in underground surgical units without electricity in Afghanistan (51).

### 4.4 Biocompatibility

Most notably, biocompatibility was not assessed in any of the included studies. This represents a fundamental translational limitation. George et al. reported that their 3DP instruments absorbed the fluid from cadavers (35). This may indicate interaction with the bodily fluid. Material selection alone is insufficient to infer biological safety, particularly where manufacturing processes may alter surface properties, porosity, or degradation behaviour. The absence of ISO (54–60) based evaluation effectively precludes clinical readiness, regardless of mechanical or functional performance. Table 6 aims to provide a structured guideline to assist the appropriate biocompatibility tests based on this standard.

**Table 6:** Proposed Pre-clinical evaluation and reporting guideline for 3D-printed surgical device for austere environments—biocompatibility.

Biocompatibility evaluation details	Explanation
Nature and type of body contact	Is the contact through skin, injured skin, mucosa or blood? Is it implantable? Details can be found in ISO 10993-1 (54)
Duration of contact	How long is the contact? Classification details can be found in ISO 10993-1
Device composition and manufacturing	What is the material before manufacture? What condition does the raw material undergo during manufacture (temperature, laser heating, etc.) Is there any additive to the main composition? Is there a post-production process that adds to or affects the device?
Relevant biological tests	Depending on the ISO 14971 (23) risk management framework, appropriate tests should be carried out. The list is denoted in ISO 10993. Below are some frequent examples; Cytotoxicity—ISO 10993-5 (55) Irritation—ISO 10993-23 (56) Sensitisation—ISO 10993-10 (57) Systemic toxicity—ISO 10993-11 (58) Genotoxicity, carcinogenicity, reproductive and developmental toxicity—ISO 10993-3 (59) Haemocompatibility—ISO 10993-4 (60)

### 4.5 Usability

Assessment of usability is a critical component in the development of novel healthcare devices, as user interaction directly impacts patient safety (61, 62). In this review, seven studies performed usability assessments. However, methodologies were highly heterogeneous. Evaluation types included Likert-scale questionnaires, timed suturing tasks, simulated procedures, and cadaveric evaluations. This inconsistency limits comparability and highlights the need for standardised usability assessment to support patient safety and research progress.

Notably, there was no application of established usability guidelines such as IEC 62366-2 (63) which guides the manufacturer to identify hazard-related scenarios and develop an appropriate evaluation plan (64). Based on this guideline, we recommend that researchers identify specific user-related hazards and evaluate by task-based usability tests. The details of each usability test are dependent upon the instrument type and its risk profile. A proposed guideline shown in Table 7 can be used to develop such tests.

**Table 7:** Proposed Pre-clinical evaluation and reporting guideline for 3D-printed surgical device for austere environment—usability.

Usability details	Explanation
Use specification	This may contain: intended medical indications, intended patient population, intended part of body or type of tissue applied to, user profile, user environment, etc. This may be obtained by contextual inquiry, observation, interview or survey.
Identify potential use errors	Manufacturers are required to identify characteristics of the user interface that could affect safety. The identification of known or foreseeable hazards. Consider listing the steps involved in using devices. This may include preparation by the scrub team, critical surgical application, and dismantling the components for reprocessing and sterilisation. Subject matter expert input may be required at this point, and working in a multi-disciplinary manner with all professionals involved is highly recommended. Consider prioritising steps that pose a high risk to patient safety.
Usability Test	Depending on the potential use error, a usability test should be documented. It should be planned to provide the data needed to evaluate the user interface. Consider testing under the most clinically realistic environment as possible to simulate situational stress.
Refine design and repeat	If the usability test is formative, the researchers should report the changes to the device and repeat the test. The final summative test should explain risk mitigation for accepted risk factors.

**Figure 10:**
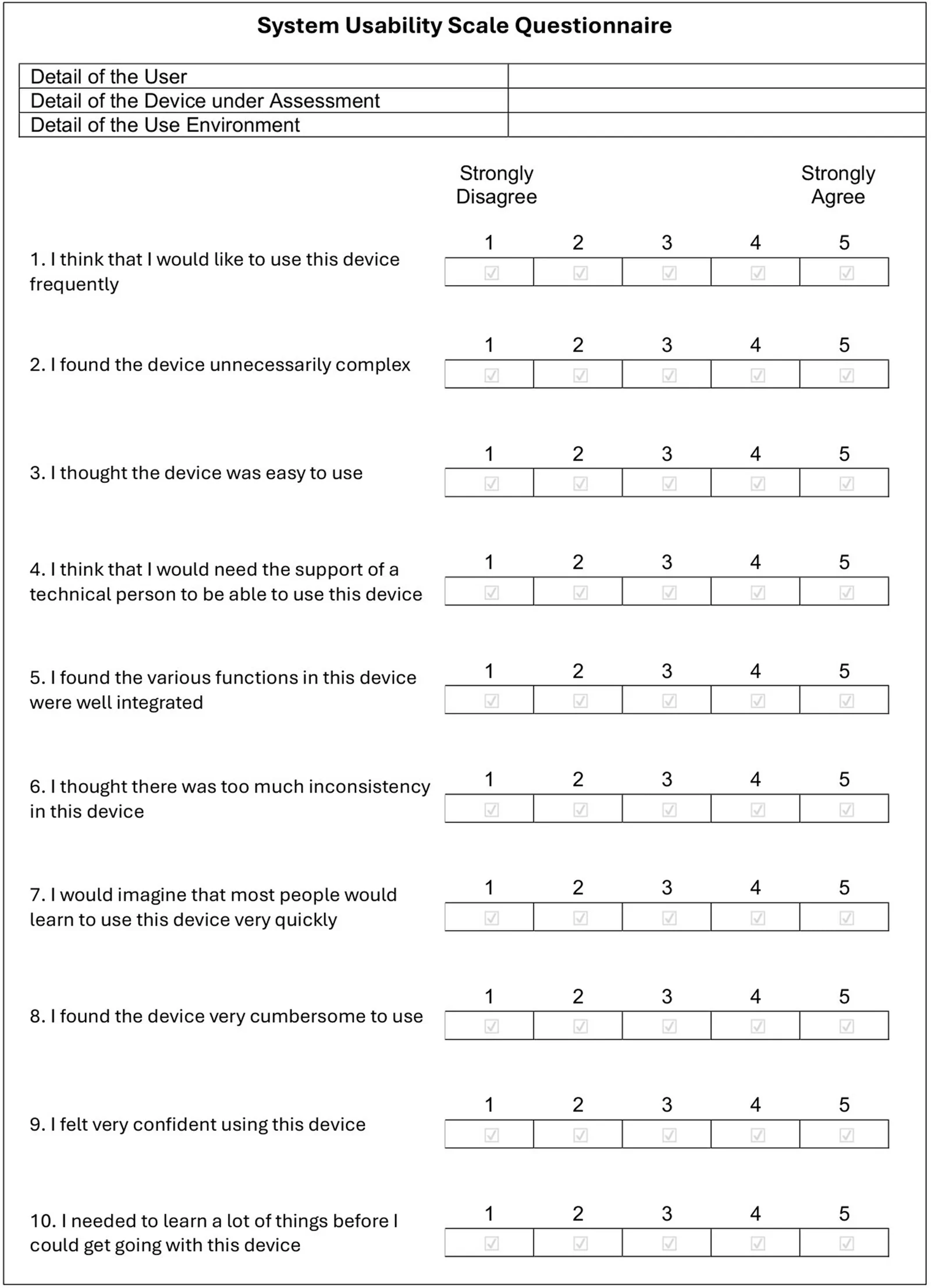
Proposed Pre-clinical evaluation and reporting guideline for 3D-printed surgical device for austere environments—system usability scale (65).

While ISO 62366-1 provides a framework for identifying and mitigating use-related risks, its application is disproportionate for the early stages of research and development. It is also impractical to implement in busy clinical environments. For 3D-printed surgical devices, where designs are iterative and rapidly evolving, the System Usability Scale (SUS) (65) offers a more pragmatic approach, enabling rapid, low-resource assessment of perceived usability and user acceptability. This allows usability feedback to be collected efficiently without interrupting clinical workflow (66, 67). Although SUS does not evaluate safety-critical use errors, it facilitates efficient comparison between design iterations and early identification of usability concerns. This supports iterative refinement prior to formal risk-based usability validation. Based on SUS, Figure 10 demonstrates an example questionnaire that researchers may utilise.

## 5 Limitations

This review has several limitations. First, although a comprehensive search strategy was employed across medical and engineering databases, the inclusion criterion requiring explicit or implied relevance to austere environments may have led to the exclusion of potentially applicable 3D-printed surgical devices not framed within this context. This may introduce selection bias. Second, the included studies were highly heterogeneous in design, device type, and outcome measures. This limited quantitative synthesis and the ability to directly compare performance across studies. Third, the evidence base was dominated by early-stage, proof-of-concept studies conducted in high-resource settings, which restricts the findings to true austere environments. Fourth, due to the absence of established quality appraisal tools for early-stage medical device development studies, a structured domain-based assessment was used in place of conventional risk-of-bias instruments. This is appropriate for this context, but this approach introduces an element of subjectivity. Finally, the relatively low number of papers identified from the search strategy may reduce confidence in the conclusion and pose a risk of publication bias. This is because unsuccessful or non-functional device designs are less frequently reported, potentially overestimating the apparent feasibility of 3D-printed surgical devices in austere settings. As the publications originate from three well-resourced English-speaking countries, there is also a risk of limited generalisability representing only a few types of austere environments. However, this may also reflect the fact that we are in the early stages of employing 3D-printed surgical devices in austere environments.

## 6 Conclusion

This review highlights the absence of standardised and comparable evaluation of novel 3D-printed surgical devices intended for austere environments across the domains recommended by the regulatory frameworks. Although some studies reported selected testing, there was substantial heterogeneity in methodologies and an absence of reproducible or comparable protocols. Manufacturing parameters were generally well described; however, most devices did not undergo rigorous mechanical testing. Established standards were largely overlooked. Where testing was attempted, such as in external fixators, protocols were often not aligned with recognised parameters. The effects of sterilisation were not assessed using standardised approaches. Biocompatibility tests were not carried out by any of the studies in this review. Usability evaluation was inconsistently performed, with significant variation in study design and outcome measures, limiting meaningful comparison across studies.

Patient safety remains paramount. As the adoption of these devices is likely to expand, there is an urgent need for structured and standardised guidance to mitigate potential risks. The framework proposed in this review seeks to standardise the evaluation and reporting of these devices in alignment with established regulatory standards. Such an approach prioritises patient safety while promoting consistency, transparency, and greater collaboration across future research and development efforts.
